# Atomic-scale observation of localized phonons at FeSe/SrTiO_3_ interface

**DOI:** 10.1038/s41467-024-47688-5

**Published:** 2024-04-23

**Authors:** Ruochen Shi, Qize Li, Xiaofeng Xu, Bo Han, Ruixue Zhu, Fachen Liu, Ruishi Qi, Xiaowen Zhang, Jinlong Du, Ji Chen, Dapeng Yu, Xuetao Zhu, Jiandong Guo, Peng Gao

**Affiliations:** 1 International Center for Quantum Materials, School of Physics, Peking University, Beijing, 100871 China; 2 Electron Microscopy Laboratory, School of Physics, Peking University, Beijing, 100871 China; 3 Department of Physics, University of California at Berkeley, Berkeley, CA 94720 USA; 4 Beijing National Laboratory for Condensed Matter Physics and Institute of Physics, Chinese Academy of Sciences, Beijing, 100190 China; 5 School of Physical Sciences, University of Chinese Academy of Sciences, Beijing, 100049 China; 6 Academy for Advanced Interdisciplinary Studies, Peking University, Beijing, 100871 China; 7 Institute of Condensed Matter and Material Physics, Frontiers Science Center for Nano-optoelectronics, School of Physics, Peking University, Beijing, 100871 China; 8 Collaborative Innovation Center of Quantum Matter, Beijing, 100871 China; 9 Interdisciplinary Institute of Light-Element Quantum Materials and Research Center for Light-Element Advanced Materials, Peking University, Beijing, 100871 China; 10 Shenzhen Institute for Quantum Science and Engineering (SIQSE), Southern University of Science and Technology, Shenzhen, 518055 China; 11 Hefei National Laboratory, 230088 Hefei, China

**Keywords:** Superconducting properties and materials, Transmission electron microscopy, Surfaces, interfaces and thin films

## Abstract

In single unit-cell FeSe grown on SrTiO_3_, the superconductivity transition temperature features a significant enhancement. Local phonon modes at the interface associated with electron-phonon coupling may play an important role in the interface-induced enhancement. However, such phonon modes have eluded direct experimental observations. The complicated atomic structure of the interface brings challenges to obtain the accurate structure-phonon relation knowledge. Here, we achieve direct characterizations of atomic structure and phonon modes at the FeSe/SrTiO_3_ interface with atomically resolved imaging and electron energy loss spectroscopy in an electron microscope. We find several phonon modes highly localized (~1.3 nm) at the unique double layer Ti-O terminated interface, one of which (~ 83 meV) engages in strong interactions with the electrons in FeSe based on ab initio calculations. This finding of the localized interfacial phonon associated with strong electron-phonon coupling provides new insights into understanding the origin of superconductivity enhancement at the FeSe/SrTiO_3_ interface.

## Abstract (web summary)

The authors characterize the phonon modes at the FeSe/SrTiO_3_ interface with atomically resolved electron energy loss spectroscopy and correlate them with accurate atomic structure in an electron microscope. They find several phonon modes highly localized at the interface, one of which engages in strong interactions with the electrons in FeSe.

## Introduction

Single unit-cell (UC) FeSe grown on SrTiO_3_ substrate has attracted strong research interest for its remarkably high superconductivity transition temperature, which is about an order of magnitude higher compared to that of bulk FeSe^1–3^. The anomalously large superconducting gap occurring only in the first UC of FeSe indicates that superconductivity is substantially enhanced by the existence of interface^1,4,5^. Replica bands were first observed in angle-resolved photoemission spectroscopy (ARPES) experiments, which have an approximately 90-to-100-meV energy shift between replica bands and main bands, despite some debate^6^, were identified as the signature of electron–phonon coupling^7–10^. Recent high-resolution electron energy loss spectroscopy (HREELS) experiments suggest that the phonons involved in electron–phonon coupling are likely to be the Fuchs–Kliewer (F–K) phonons of SrTiO_3_^11–13^. The similarity between the energy of the F–K phonon and the energy shift of replica bands indicates that this phonon could contribute predominately to the electron–phonon coupling.

The emergence of localized phonons at the interface is widely understood to be a consequence of the breakdown of translational symmetry, which alters the local bonds and, subsequently, the lattice vibrations at the interface. However, precisely probing the highly localized phonons across the FeSe/SrTiO_3_ interface is challenging for surface analysis techniques such as ARPES and HREELS due to their large probe size and their unique setup configurations. On the other hand, the lack of accurate knowledge on the atomic structure of interface in previous studies blurs our understanding of the structure–property relation. It can be expected that the interface properties, including the electronic structures, phonon modes, and electron–phonon coupling, strongly depend on the atomic structure. In fact, the atomic structure of this interface is sensitive to sample history, such as annealing and surface treatment during sample preparation^14,15^. Theoretically, previous investigations either approximated the phonon structure without providing an accurate depiction of the atomic structure^16–18^ (relying solely on the normal monolayer Ti–O termination, which, however, does not align well with experimental observations^14,19,20^), or they concentrated primarily on the electron bands at the interface^21–23^. The variable and complicated atomic structure of the interface poses challenges for ab initio calculations aiming to meticulously reproduce or predict interfacial properties such as the phonon structure or electron–phonon coupling. To date, the localized phonons across the FeSe/SrTiO_3_ interface are still largely unknown, not to mention precisely correlating with their atomic arrangements or superconductivity properties, which motivates our present study.

In this work, we study the FeSe/SrTiO_3_ interface by using scanning transmission electron microscopy-electron energy loss spectroscopy (STEM-EELS). The cutting-edge developments in STEM-EELS have made it possible to directly image phonon excitations at the nanoscale credit to its high spatial and energy resolution^24–28^, which is very suitable for the study of interfacial phonons in heterostructures^29–33^. Meanwhile, the ability to reveal the atomic structure, electronic state, and phonon mode allows us to understand their interrelationships and thus the underlying mechanism.

We first combine high-angle annular dark-field (HAADF) images with atomically resolved core-loss spectra to recognize the atomic structure of double-layer Ti–O termination at the FeSe/SrTiO_3_ interface. The structure reconstruction exists in the top layer Ti–O (the top layer is defined as a layer adjacent to FeSe). From the atomically resolved phonon spectra across the interface, highly localized interfacial phonon modes can be observed. The ab initio calculations of interfacial phonons by taking subsistent double Ti–O termination layer into consideration further confirm that the interfacial phonons originated from the double layer Ti–O termination, i.e., phonons with energy ~18 and ~81 meV are centered at the top layer, and phonons with energy ~51 and ~80 meV are centrally enhanced at the bottom layer. Particularly, we identify a strongly coupling interfacial (SCI) phonon mode (~83 meV) at the double layer Ti–O termination. This mode can promote pronounced electron–phonon coupling at the interface. Thus, such a localized interfacial phonon associated with short-range interactions likely plays a pivotal role in the enhancement of interface superconductivity, complementary to the previously reported F–K phonon mode with long-range interactions. Our atomic-scale measurements of phonons across the FeSe/SrTiO_3_ interface and studies of correlation among the atomic structure, phonon structure, and quantum properties help us to understand the past experiments and provide new insights into the mechanism of the enhanced interfacial superconductivity.

## Results

### Interface atomic structure characterizations

**Fig. 1:**
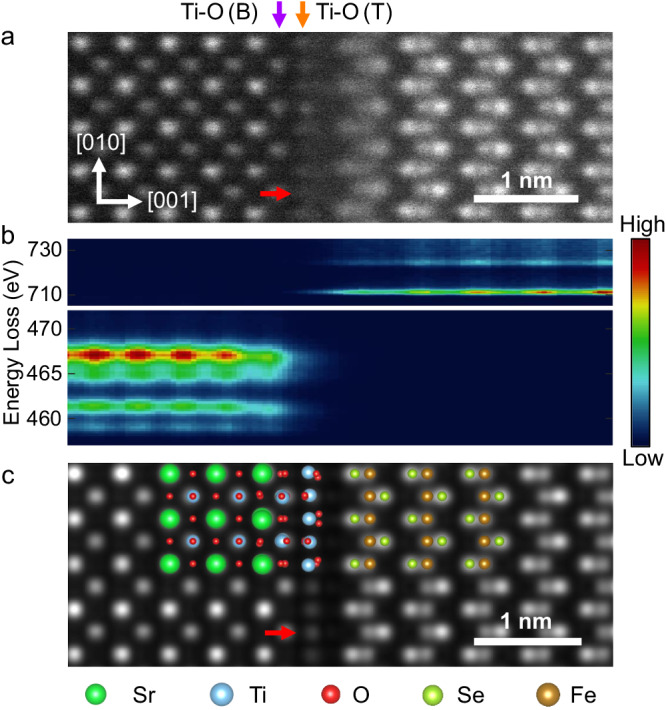
The atomic structure of the FeSe/SrTiO_3_ interface. **a** A high-angle annular dark-field (HAADF) image of FeSe/SrTiO_3_ interface from [100] zone axis. The purple and orange arrows indicate the bottom Ti–O(B) and top Ti–O(T) layers, respectively. **b** The core-loss of Fe–L_2,3_ edge (upper panel) and Ti–L_2,3_ edge (lower panel) across the interface. **c** The simulated HAADF image and overlaid atomistic model. The red arrows in **a** and **c** point to the extra atom contrast between the ordinary top Ti–O column.

In our study, the substrate treatment, sample growth, and annealing procedure are exactly the same as previously reported conditions that are optimal for superconductivity^34^. We first study the atomic structure of the FeSe/SrTiO_3_ interface by atomically resolved STEM-HAADF and core-level EELS. Figure 1a is a HAADF image of the FeSe/SrTiO_3_ interface viewed along the [100] zone axis. The double layer Ti–O termination is observed, in accordance with former researches^14,19,20^. The bottom/top layers of double-layer Ti–O termination are marked in Fig. 1a with purple/orange arrows and named Ti–O(B)/Ti–O(T) in the following text. The existence of double layer Ti–O termination can also be confirmed from the atomically resolved core-loss EELS of Fe–L_2,3_ edge (upper panel) and Ti–L_2,3_ edge (lower panel) in Fig. 1b. Changes of Ti–L_2,3_ edge at Ti–O(B) and Ti–O(T) layers are attributed to the distortion of TiO_6_ octahedron and possible oxygen vacancies introduced during sample annealing^35,36^(see Fig. S1 for better visualization). In the Ti–O(T) layer, there is an extra atom contrast between the ordinary top Ti–O column (red arrow in Fig. 1a), which is expected to be oxygen atom position and invisible in the HAADF image. This has been reported and explained as the reconstructed Ti–O termination layer^23^, e.g., recent studies revealed $\sqrt{13}\times \sqrt{13}{{{{{\rm{R}}}}}}33.7^\circ$ reconstruction of the Ti–O layer after FeSe was grown^20,22^. In fact, various SrTiO_3_ surface reconstructions with additional Ti–O layers have been reported^37–39^. To verify the assumption that the extra atom contrast comes from reconstruction, we performed the relaxation with density functional theory (DFT) on FeSe/SrTiO_3_ structure without reconstruction, and with $\sqrt{2}\times \sqrt{2}{{{{{\rm{R}}}}}}45^\circ$,$\,\sqrt{5}\times \sqrt{5}{{{{{\rm{R}}}}}}26.6^\circ$,$\,\sqrt{10}\times \sqrt{10}{{{{{\rm{R}}}}}}18.3^\circ$, $\sqrt{13}\times \sqrt{13}{{{{{\rm{R}}}}}}33.7^\circ$ reconstruction, and corresponding STEM-HAADF image simulations using QSTEM^40^ (see Fig. S2 for details). From the simulation, the structure without reconstruction does not show extra atom contrast on the red-arrow-pointed site. Although the $\sqrt{2}\times \sqrt{2}{{{{{\rm{R}}}}}}45^\circ$ structure does show extra atom contrast, the distance between the bottom Se layer and the top Ti layer is significantly smaller than the experimental result. The other three structures are sub-structures of $\sqrt{13}\times \sqrt{13}{{{{{\rm{R}}}}}}33.7^\circ$ reconstruction^39^, and their simulated images all agree well with the experiment. Due to the reported experimental evidence of$\,\sqrt{5}\times \sqrt{5}{{{{{\rm{R}}}}}}26.6^\circ$ reconstruction under similar annealing conditions^41^ (see Methods for detail), we pick$\,\sqrt{5}\times \sqrt{5}{{{{{\rm{R}}}}}}26.6^\circ$ reconstruction as a rational structure to explain our experimental result. Its simulated HAADF image and atomistic models are shown in Fig. 1c.

### Atomically resolved phonon spectra across the FeSe/SrTiO_3_ interface

**Fig. 2:**
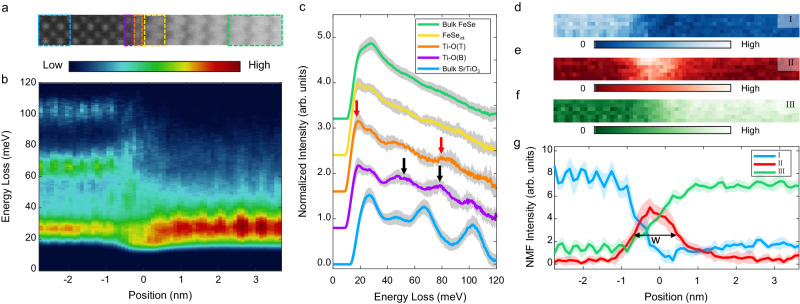
The atomically resolved phonon spectra at the FeSe/SrTiO_3_ interface. **a** The HAADF image showing the region where EELS spectra are acquired. **b** The map of atomically resolved off-axis EELS spectra across the interface. **c** The spectra extracted from bulk SrTiO_3_ (blue), Ti–O(B) (purple), Ti–O(T) (orange), FeSe layer adjacent to the interface (yellow), and bulk FeSe (green). Their spatial regions are labeled by dashed rectangles with corresponding colors in (**a**). The red and black arrows point to the main features that are enhanced at Ti–O(T) and Ti–O(B) layers, respectively. The gray shades are the standard deviations. **d**, **e** The non-negative matrix factorization (NMF) intensity map for a component I (Ti–O in SrTiO_3_, **d**), component II (interface, **e**), and component III (FeSe and Sr in SrTiO_3_, **f**). **g** The line profile of NMF maps across the interface. The colored shades are corresponding standard deviations. The full-width-at-half-maxima for component II is labeled as w in the figure.

We then measure phonon spectra across the FeSe/SrTiO_3_ interface by atomically resolved STEM-EELS. The HAADF image of the acquisition region and a line profile of corresponding EEL spectra are shown in Fig. 2a and Fig. 2b, respectively. The clear contrast between the Sr column and Ti–O column demonstrates sufficiently high spatial resolution to distinguish interfacial phonon and the rationality of column-by-column spectra analysis. As shown in Fig. 2b, the SrTiO_3_ transverse optical (TO) branch phonon around 65 meV splits into two branches as approaching the interface from the SrTiO_3_ side, a transformation attributable to the emergence of the interfacial phonon. One of the phonon branches experiences a blue shift, positioning itself within the energy gap of 65–100 meV, while the other transitions toward lower energy domains. For better visualization, the spectra extracted from bulk SrTiO_3_ (blue), Ti–O(B) (purple), Ti–O(T) (orange), FeSe layer adjacent to the interface (FeSe_int_, yellow), and bulk FeSe (green) are shown in Fig. 2c. Their spatial regions are labeled by dashed rectangles with corresponding color in Fig. 2a. We find that several local interfacial phonon modes emerge due to the presence of double layer Ti–O termination. Even with broadening over adjacent atomic layers, we have still successfully pinpointed the localization centers of these modes. The red arrows in Fig. 2c point to the spectral features that show enhancement centered at the Ti–O(T) layer, whose energies are ~18 and ~81 meV. The black arrows point to the features that are centrally located at the Ti–O(B) layer, whose energies are ~51 meV and ~80 meV.

To better separate the intrinsic spectra from the interface, we applied non-negative matrix factorization (NMF) on the whole spectrum image. NMF is a powerful tool to provide well-interpretable characteristics of data, which has already been successfully applied to the identification of EEL spectra^42,43^. Three components are found to best describe the acquired data. Their intensity maps are shown in Fig. 2d–f. Figure 2g shows the line profile of their intensity map across the interface. Component I shows atomic contrast consistent with the Ti column in HAADF and decays fast as it approaches the interface, indicating its origin from the vibration of the Ti–O plane in SrTiO_3_. Component III shows the contrast of the Sr atom and FeSe column, thus is attributed to the sum of vibration signals from FeSe and Sr atom in SrTiO_3_, considering their similarity in vibration energy. Intriguingly, component II is highly localized at the interface, constructing a peak centered at the Ti–O(T) layer, spreading over the double layer Ti–O termination and the first FeSe layer. This helps to explain the large enhancement of superconductivity only occurring in the first UC of FeSe. The full-width-at-half-maxima (FWHM) of component II (w in Fig. 2g) is ~1.3 nm. A different and independent approach to extracting the features of interfacial phonons is finding the minimum difference between the measured spectrum and a possible linear combination of two bulk spectra. The result is shown in Fig. S3, in which the width ~1.3 nm agrees well with the NMF result, further confirming the presence and highly localized nature of interfacial phonons.

Notably, the feature above 45 meV in FeSe spectra still remains even far from the interface, exceeding the max frequency of pure FeSe^17^. Therefore, it must come from the SrTiO_3_ substrate. This can be found in the NMF spectrum of component III as well (Fig. S4a). We ascribe these signals to the phonon polaritons (PPs), i.e., the F–K phonons of SrTiO_3_, which are found to penetrate into FeSe in previous works^11–13^. To confirm this, we fitted the spectra of NMF component III and compared it to the result that was acquired under the on-axis experimental setup. The similarity of spectrum shape and fitted energy between on-axis and off-axis results support our assumption (see Fig. S4b–d for detail).

### Electron–phonon coupling of interfacial phonons

**Fig. 3:**
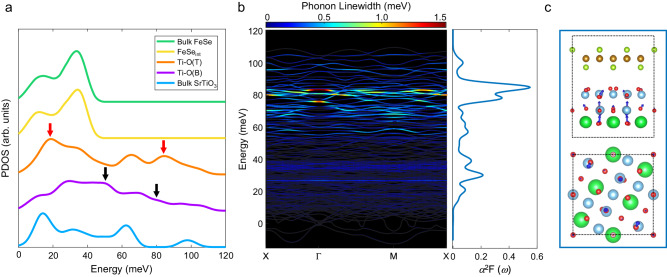
The calculated phonon DOS, dispersion, and electron–phonon coupling at FeSe/SrTiO_3_ interface. **a** The calculated phonon density of states (PDOS) extracted from bulk SrTiO_3_ (blue), Ti–O(B) in the interface structure(purple), Ti–O(T) in the interface structure (orange), FeSe layer in the interface structure (yellow) and bulk FeSe (green). The red and black arrows point to the main features that are enhanced at Ti–O(T) and Ti–O(B) layers, respectively. **b** Phonon linewidths due to electron–phonon coupling mapped on phonon dispersion and the Eliashberg spectral function at Γ point for calculated structure. The phonon with energy ~83 meV has the strongest coupling strength with FeSe electrons at Γ point, i.e., strongly coupling interfacial (SCI) mode. **c** The side view (upper panel) and top view (lower panel) of phonon eigenvectors of SCI mode in calculated interface structure.

To get further insights into the interfacial phonon, we carried out ab initio phonon calculations on an interface model of FeSe on the double-layer Ti–O terminated SrTiO_3_ (see Methods). Corresponding to the experimental spectra, the projected phonon density of states (PDOS) of bulk SrTiO_3_, Ti–O(B) layer, Ti–O(T) layer, FeSe_int_ layer, and bulk FeSe are plotted in Fig. 3a. Similarly, the spectrum features ~18 meV and ~81 meV that are significantly enhanced at Ti–O(T) layer (red arrows), and spectrum features ~51 meV and ~80 meV that are enhanced at Ti–O(B) layer (black arrows), are spotted. The eigenvectors of these modes from side view and top view are illustrated in Extend Data Fig. 5a-d. These modes involve vibrations much stronger at either Ti–O(B) or Ti–O(T) layer, indicating their highly localized nature (more details in Fig. S5e–h). The projected dispersions of interface models further demonstrate the localized nature of interfacial phonons and dynamical stability of the reconstructed structure (Fig. S6), i.e., significant imaginary frequency only exists in the reconstruction-free interface model. All these characters agree well with the experimental findings despite a small mismatch of energy that is within the accuracy of the DFT calculations.

To establish a direct correlation between observed interfacial phonons and enhanced superconductivity, we extracted the electron–phonon coupling features from the calculations. The phonon linewidth due to electron–phonon coupling mapped on the phonon dispersion and the Eliashberg spectral function at Γ point for calculated structure is shown in Fig. 3b. Particularly, we find that an SCI phonon mode which has the strongest coupling strength in our calculations. Firstly, it has an energy of ~83 meV and maximum line width among all modes at Γ point, which corresponds to the peak of the same energy in the Eliashberg spectral function. From the Eliashberg spectral function curves, we obtain the electron–phonon coupling constant^44^ of the SCI phonon mode as *λ* ≈0.10. This suggests that interfacial modes may play a significant role in the interfacial electron–phonon coupling, complementary to the previously considered optical modes of SrTiO_3_ or F–K phonons. Secondly, its eigenvector from the side and top view is shown in Fig. 3c, suggesting this mode is caused by the out-of-phase vibration of Ti and O ions. Fig. S7 demonstrates the localized nature of this SCI mode. Such vibrations induce a dipole and change the electric field at the interface, leading to interactions with electrons in FeSe^7^. Thirdly, the electron–phonon coupling usually leads to the phonon softening^45^. To confirm this behavior in our system, we performed calculations with a double-layer Ti–O terminated SrTiO_3_ surface model (without FeSe). Distinctively, we find a mode analogous to SCI mode with respect to vibration eigenvector (see Fig. S8). This model has an energy of ~93 meV at the FeSe-free SrTiO_3_ surface, which is about 10 meV higher than the energy of the SCI mode. The pronounced softening in energy for the same vibrational mode can solely be attributed to the presence of FeSe. The substantial phonon softening in this system implies the presence of strong electron–phonon coupling induced by the observed SCI mode, i.e., the phonons of SrTiO_3_ strongly interact with the electrons in FeSe.

## Discussion

In previous studies, F–K phonons from the SrTiO_3_ substrate have usually been regarded as the primary origin of interfacial electron–phonon coupling in this system^11–13^. The intense long-range dipolar field generated by the substrate F–K phonons has been widely believed to enhance the electron pairing in FeSe, although the exact microscopic interaction mechanism is still awaiting clarification. Our results show that besides the F–K phonons with long-range interactions, the localized interfacial phonons with short-range interactions also make unique contributions to the electron–phonon coupling, thereby can interact in complementary to F–K phonons and enhancing the electron–phonon coupling at the interface collaboratively. Our results provide new insights into understanding the underlying mechanisms of enhanced superconductivity at interfaces.

It should be noted, however, that there are some limitations to our present study, and further investigations are needed in the future to more precisely correlate the localized interfacial phonons with the superconductivity property. For the calculation, although our model is already$\,\sqrt{5}\times \sqrt{5}{{{{{\rm{R}}}}}}26.6^\circ$ reconstructed, the calculated electron–phonon coupling strength of the SCI mode is likely overestimated. To more accurately estimate the electron–phonon coupling strength, an even denser q-grid is probably required^18^. Furthermore, some other effects, such as magnetic effect^17^, charge transfer^46^, or correlation^47^, that may further enhance the coupling strength also need to be considered in the calculations. For the experiments, tracking the evolution of local interfacial phonons during the superconducting transition by using in situ transport measurement in the scanning transmission electron microscope would allow us to directly correlate the local interfacial phonons with the superconducting properties, thus unambiguously revealing the role of local interfacial phonons. Therefore, our study will likely stimulate further theoretical and experimental studies in the future.

In summary, we carried out direct measurements of phonon spectra at the FeSe/SrTiO_3_ interface and correlated the features with the unique double-layer Ti–O termination with reconstruction. We implemented the ab initio calculations of interfacial phonons by taking the subsistent double Ti–O termination layer into consideration, which agrees well with the experiment. We find that highly localized phonons are emergent at the interface, which promotes intense electron–phonon coupling. These findings take an essential step towards revealing the role of interface in the interface-enhanced superconducting systems.

## Methods

### Sample growth and TEM sample preparation

A FeTe(20 UC) /FeSe(20 UC)/SrTiO_3_ structure was grown by molecular beam epitaxy. Before the epitaxial growth of FeSe films, the Nb-doped (0.5 wt%) SrTiO_3_ (001) substrates (from Shinkosha Co. Ltd) were pretreated in ultrahigh vacuum (UHV) at 1000 °C for 45 min to obtain a Ti–O plane terminated surface. The high-quality FeSe film was grown by co-depositing high-purity Fe (99.99%) and Se (99.99 + %) with a flux ratio of ~1:10 onto treated SrTiO_3_ held at 470 °C. After growing FeSe, the sample was annealed at 480 °C for 3 h under UHV. Afterward, the FeTe capping layer was grown by co-depositing Fe and Te with a flux ratio of ~1:5 onto FeSe held at 385 °C. The cross-sectional TEM sample was prepared by the ThermoFisher Helios G4 UX Focused Ion Beam (FIB) system.

### TEM characterization and EELS data acquisition

The STEM-HAADF images shown in Fig. 1a, Fig. S1b, and Fig. S2a were recorded using an aberration-corrected FEI Titan Themis G2 operated at 300 kV. The beam convergence semi-angle was 30 mrad and the collection angle was 39-200 mrad. The HAADF image shown in Fig. 2a was recorded at 60 kV using a Nion U-HERMES200 microscope equipped with both a monochromator and aberration correctors, with 35-mrad convergence semi-angle and 80–200 mrad collection semi-angle. All the EELS data were acquired on the same Nion U-HERMES200 microscope operated at 60 kV, with 35-mrad convergence semi-angle and 24.9-mrad collection semi-angle. EELS data shown in Fig. 1b, Fig. S1a, Fig. S1c, and Fig. S4c, d was collected under the on-axis experimental setup namely, the center of the collection aperture was placed at the center of the direct beam disk. Data shown in Fig. 1b and Fig. S1a was recorded with 128×12 pixels within the range of 6 nm across the interface. The energy dispersion was set as 0.262 eV/channel. Data shown in Fig. S4d, c was collected with 128×16 pixels within the range of 8 nm across the interface. The energy dispersion was 0.5 meV/channel. Data shown in Fig. 2b–g, Fig. S3, and Fig. S4a, b was collected under the off-axis experimental setup, in which the electron beam was displaced from the optical axis along the [010] direction of SrTiO_3_ with 60 mrad to greatly reduce the contribution from the long-range dipole scattering^28,48^. The data was collected with 80×10 pixels within the range of 8 nm across the interface. The energy dispersion was 0.5 meV/channel.

### EELS data processing

All acquired EEL spectra were processed by custom-written MATLAB code^49^. The EEL spectra were first aligned by their normalized cross-correlation. Subsequently, the spatial drift correction was applied to obtain line-scan data. The background of the core-loss EELS data shown in Fig. 1b and Fig. S1a–c was fitted by power-law and then subtracted from the whole space. The phonon spectra shown in Fig. 2b–g, Fig. S3, and Fig. S4 were multiplied by the square of energy rather than fitting with a background function to resolve the difficulty of fitting the background function at low energy and to treat spectra in different spatial components uniformly. This method has already been performed effectively in processing EELS data acquired in SrTiO_3_ and other analogous materials^33^. Lucy–Richardson deconvolution was then employed to ameliorate the broadening effect caused by the finite energy resolution. NMF was performed to decompose the off-axis data. NMF is a computational method that decomposes a non-negative matrix into the product of two non-negative matrices, often used for feature extraction^50^. We found 3 components that best describe our data. The spectra in Fig. S4 were fitted by Gaussian peak. In Figure S3, the interface component of the spectra was extracted by fitting measured spectra with a linear combination of SrTiO_3_ spectrum and FeSe spectrum. The fitting was performed by minimizing ||$S\left(\omega \right)-{a}_{1}{S}_{{{{{{\rm{SrTi}}}}}}{{{{{{\rm{O}}}}}}}_{3}}\left(\omega \right)-{a}_{2}{S}_{{{{{{\rm{FeSe}}}}}}}\left(\omega \right)$|| while keeping the residual non-negative, where $S\left(\omega \right)$ is the measured spectrum in Fig. 2b, ${S}_{{{{{{\rm{SrTi}}}}}}{{{{{{\rm{O}}}}}}}_{3}}$ means the bulk SrTiO_3_ spectra, ${S}_{{{{{{\rm{FeSe}}}}}}}$ means the bulk FeSe spectra, and ${a}_{1}$, ${a}_{2}$ are adjusted coefficients.

### Ab initio calculations

Density functional theory calculations were performed using Quantum ESPRESSO^51,52^ with the Perdew-Burke-Ernzerhof for solid (PBEsol) exchange-correlation functional^53^ and the projector augmented wave (PAW) method pseudopotential^54^. The kinetic energy cut-off was 60 Ry for wavefunctions and 600 Ry for charge density and potential. The reconstruction-free, $\sqrt{2}\times \sqrt{2}{{{{{\rm{R}}}}}}45^\circ$ reconstruction,$\,\sqrt{5}\times \sqrt{5}{{{{{\rm{R}}}}}}26.6^\circ$ reconstruction,$\,\sqrt{10}\times \sqrt{10}{{{{{\rm{R}}}}}}18.3^\circ$ reconstruction and $\sqrt{13}\times \sqrt{13}{{{{{\rm{R}}}}}}33.7^\circ$ reconstruction FeSe/SrTiO_3_ slab structures containing 1 UC FeSe connected to the double-layer Ti–O terminated 3 UC SrTiO_3_ were built, for which an in-plane lattice constant of ${a}_{{{{{{\rm{SrTi}}}}}}{{{{{{\rm{O}}}}}}}_{3}}=3.893\mathring{\rm A}$ (the optimized lattice constant of bulk SrTiO_3_ under the used exchange-correlation functional and pseudopotential) and an out-of-plane lattice constant of $c=40\mathring{\rm A}$ was chosen. The total atom numbers of these structures are 22, 45, 110, 217, and 277, respectively. The lattice mismatch between SrTiO_3_ and FeSe was ignored. All the structures were optimized while keeping the lattice constant invariant until the residual force was below 10^−5^ Ry/Bohr on every atom and the total energy gradient below 10^−10^ Ry, reaching the numerical limit of the software.

For the phonon calculation, we used FeSe (1 UC)/SrTiO_3_(3 UC) interface models. Only reconstruction-free and$\,\sqrt{5}\times \sqrt{5}{{{{{\rm{R}}}}}}26.6^\circ$ reconstruction FeSe/SrTiO_3_ structures are applied, because the $\sqrt{2}\times \sqrt{2}{{{{{\rm{R}}}}}}45^\circ$ reconstruction structure is not consistent with our experimental observation, and $\sqrt{10}\times \sqrt{10}{{{{{\rm{R}}}}}}18.3^\circ$ reconstruction or $\sqrt{13}\times \sqrt{13}{{{{{\rm{R}}}}}}33.7^\circ$ reconstruction structures contain too many atoms to be calculated in the DFT framework. The dynamical matrices and force constants were obtained using Phonopy^55^. The treatment of non-analytical terms is implemented in the Quantum Espresso package. The projected phonon DOS (PDOS) was calculated by interpolating the dynamical matrix on a 15 × 15 × 5 q-mesh. No evident change in PDOS can be observed in the SrTiO_3_ 2 UC away from the interface compared with bulk SrTiO_3_, manifesting that our model is large enough to distinguish the interfacial SrTiO_3_ from the bulk SrTiO_3_. The calculated PDOS contains small imaginary frequencies occupying ~1% of the total PDOS of SrTiO_3_ along with the double-layer Ti–O termination, close to that obtained from the calculation in bulk SrTiO_3_ performed in the same way. However, the imaginary frequencies did not appear in the region around the Γ point, thus not confounding the analysis of phonon modes at the Γ point. The eigenvectors in Fig. 3c, Fig. S5, S7, and S8 are picked at Γ point. As a comparison, a surface structure only containing 3 UC double layer Ti–O terminated SrTiO_3_ was relaxed separately and then used for phonon calculation. A higher-energy surface mode ~93 meV was found, whose eigenvector resembles that of the SCI mode. As another comparison, phonon calculation of fully relaxed reconstruction-free FeSe/SrTiO_3_ structure was performed using both the FD method with a 2 × 2 × 1 supercell and the DFPT method with a 2 × 2 × 1 q-mesh. No difference is found between the results of the two methods. Its projected phonon dispersion is shown in Fig. S6b. Several phonon modes with large imaginary frequencies emerge at Γ point, indicating the dynamical instability of the structure.

To confirm the spatial characteristics of the SCI mode, we examined all phonon modes in the interface model containing 3 UC SrTiO_3_. The calculation result also shows the presence of this SCI mode, which only involves atoms of the top layer of SrTiO_3_, consolidating that this mode is highly localized at the interface (see Fig. S7).

For the phonon calculation of bulk SrTiO_3_ and bulk FeSe in Fig.3a, we built the conventional one-unit-cell SrTiO_3_ and FeSe and fully relaxed with constraint of in-plane lattice constant $a=3.893\mathring{\rm A}$. The interatomic forces are calculated by DFPT with a 4 × 4 × 4 q-mesh. The PDOS was calculated by diagonalizing the dynamical matrix interpolated on a 20 × 20 × 20 q-mesh and projected onto all the atoms in the unit cell.

For the electron–phonon coupling calculation, a $\,\sqrt{5}\times \sqrt{5}{{{{{\rm{R}}}}}}26.6^\circ$ reconstructed FeSe/SrTiO_3_ slab structure containing 1 UC FeSe connected to the double-layer Ti–O terminated 1 UC SrTiO_3_ (60 atoms in total) was applied using density functional perturbation theory (DFPT) implemented in Quantum ESPRESSO. We chose 1 UC SrTiO_3_ instead of 3 UC (110 atoms in total) for electron–phonon coupling calculation due to the limitation of computing resources. As a comparison, the phonon structure corresponding to this model was also calculated using the finite displacement method with a combination of Phonopy and Quantum ESPRESSO using a 1 × 1 × 1 supercell. No evident change in phonon structure was observed in the results of the DFPT or FD method for this structure. A dense mesh of 16 × 16 × 4 k-points was used for the sum of electron–phonon coefficients at the Fermi energy. Other parameters are kept the same as in the above text.

### Supplementary information

Supplementary Information

Peer Review File

## Data Availability

All data generated in this study have been deposited in the Open Science Framework database under the accession code https://osf.io/dt8ej/.

## Code availability

The code used in this study for the EELS data processing and calculation result post-processing has been deposited in the Zenodo database.
