# Supplementary material for: Atomic-scale observation of localized phonons at FeSe/SrTiO3 interface
Source: Nat Commun. 2024 Apr 23;15:3418. doi: 10.1038/s41467-024-47688-5 (PMC11039677; doi:10.1038/s41467-024-47688-5)
Supplement: Supplementary file 1 — Supplementary Information [file 41467_2024_47688_MOESM1_ESM.pdf]

Supplementary information for
Atomic-scale observation of localized phonons at FeSe/$SrTiO_3$ interface

Ruochen Shi[1,2#], Qize Li[1,2,3#], Xiaofeng Xu[4,5#], Bo Han[1,2], Ruixue Zhu[1,2], Fachen Liu[2,6], Ruishi Qi[3], Xiaowen Zhang[1,2], Jinlong Du[2], Ji Chen[7,8,9], Dapeng Yu[2,10,11], Xuetao Zhu[4,5]*, Jiandong Guo[4,5]*, Peng Gao[1,2,8,9,11]*

[1] International Center for Quantum Materials, School of Physics, Peking University, Beijing 100871, China

[2] Electron Microscopy Laboratory, School of Physics, Peking University, Beijing 100871, China

[3] Department of Physics, University of California at Berkeley, Berkeley, CA 94720, USA

[4] Beijing National Laboratory for Condensed Matter Physics and Institute of Physics, Chinese Academy of Sciences, Beijing 100190, China

[5] School of Physical Sciences, University of Chinese Academy of Sciences, Beijing 100049, China

[6] Academy for Advanced Interdisciplinary Studies, Peking University, Beijing 100871, China.

[7] Institute of Condensed Matter and Material Physics, Frontiers Science Center for Nano-optoelectronics, School of Physics, Peking University, Beijing 100871, China.

[8] Collaborative Innovation Center of Quantum Matter, Beijing 100871, China

[9] Interdisciplinary Institute of Light-Element Quantum Materials and Research Center for Light-Element Advanced Materials, Peking University, Beijing 100871, China

[10] Shenzhen Institute for Quantum Science and Engineering (SIQSE), Southern University of Science and Technology, Shenzhen 518055, China

[11] Hefei National Laboratory, 230088 Hefei, China

[#] *R. Shi, Q. Li, and X. Xu contributed equally to this work.*

* *Corresponding author. E-mail: xtzhu@iphy.ac.cn, jdguo@iphy.ac.cn, pgao@pku.edu.cn*

Supplemental Figures

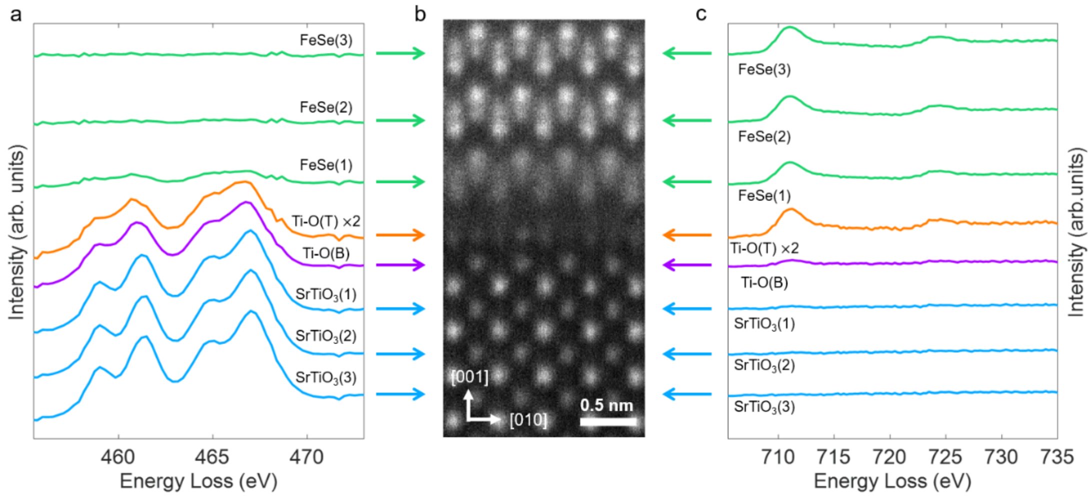

Fig. S1. The core-loss spectra of Ti-$L_{2,3}$ edge and Fe-$L_{2,3}$ across the FeSe/$SrTiO_3$ interface. a. The core-loss spectra of Ti-$L_{2,3}$ edge extracted from $SrTiO_3$ that is 1-3 UC away from the interface (blue), Ti-O(B) (purple), Ti-O(T) (orange), and FeSe that is 1-3 UC away from the interface (green). The spectra are offset, and the spectra of Ti-O(T) is multiplied by 2 for clarity. Four peaks representing the t_{2g}-e_g splitting of Ti-$L_{2,3}$ edges are clearly visible. The peaks are broader and the splitting is less pronounced at Ti-O(B) and Ti-O(T) layers, indicating a local change of electron states at the interface. **b.** The HAADF image showing the region where the core-loss data are acquired. The color arrows point to the atom layers corresponding to the region where the spectra are extracted. **c.** The core-loss spectra of Fe-$L_{2,3}$ edge extracted from $SrTiO_3$ that is 1-3 UC away from the interface (blue), Ti-O(B) (purple), Ti-O(T) (orange), and FeSe that is 1-3 UC away from the interface (green). The spectra are offset, and the spectra of Ti-O(T) is multiplied by 2 for clarity. We note that the Fe core-loss signal is less localized in the Fe columns next to the Se columns. The possible reasons include the roughness of STO surface (atomic step of surface along the observation direction), the intrinsic delocalization effect, or the mixing of Fe-Se.

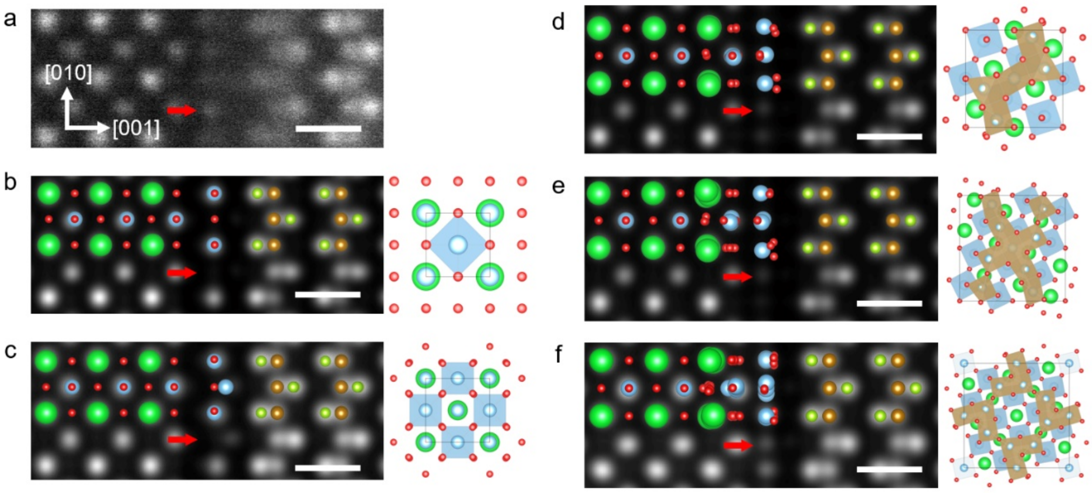

Fig. S2. The experimental and simulated HAADF images of various FeSe/$SrTiO_3$ interface structures. a. Enlarged experimental HAADF image of FeSe/$SrTiO_3$ interface viewed from [100] zone axis. **b – f.** Simulated HAADF image overlaid by atomistic model from side view (left panel) and atomistic model from top view (right panel) of $SrTiO_3$ surface with no reconstruction (**b**), $\sqrt{2} \times \sqrt{2}$ R45° reconstruction (**c**), $\sqrt{5} \times \sqrt{5}$ R26.6° reconstruction (**d**), $\sqrt{10} \times \sqrt{10}$ R18.4° reconstruction (**e**) and $\sqrt{13} \times \sqrt{13}$ R33.7° reconstruction (**f**). The blue polyhedrons in top views are complete TiO_6 octahedrons, while brown polyhedrons are TiO_5 tetrahedrons. The red arrow in each panel point to the extra atom contrast between ordinary top Ti site. Scale bar is 0.5 nm.

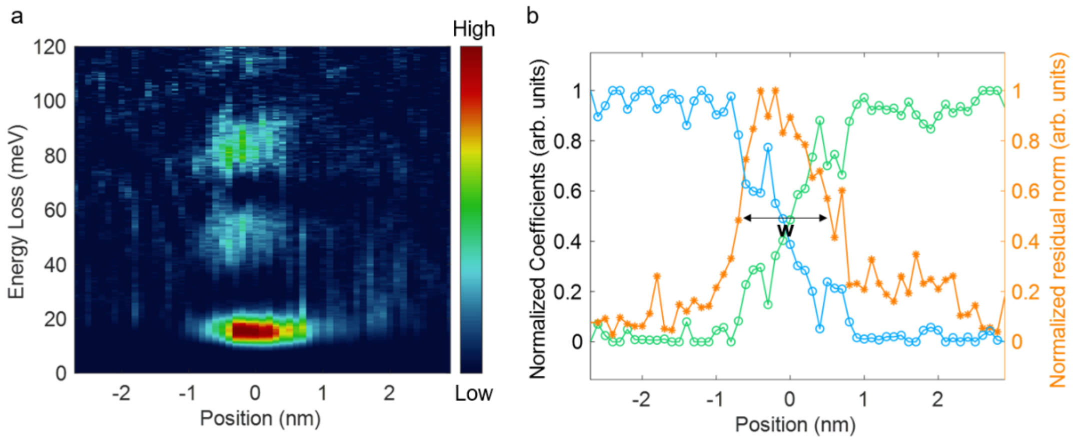

Fig. S3. The interface component of the spectra extracted by fitting measured spectra with linear combination of $SrTiO_3$ spectrum and FeSe spectrum. The fitting was performed by minimizing $\left\|S(\omega)-a_1 S_{\mathrm{SrTiO}_3}(\omega)-a_2 S_{\mathrm{FeSe}}(\omega)\right\|$ while keeping the residual non-negative, where $S(\omega)$ is the measured spectrum (Fig. 2b), S_{SrTiO_3} means the bulk $SrTiO_3$ spectra, S_{FeSe} means the bulk FeSe spectra, and a_1, a_2 are adjusted coefficients. **a.** The fitting residual, which represents the intensity of newly emergent interfacial phonons. The match between fitting result and NMF result proves the self-consistency of the data and analysis process. **b.** The line profile of normalized fitting coefficients from bulk $SrTiO_3$ (blue) and bulk FeSe (green), and the normalized residual norm (orange). Again, the FWHM of residual norm w is ~1.3 nm, in excellent agreement with the result of NMF.

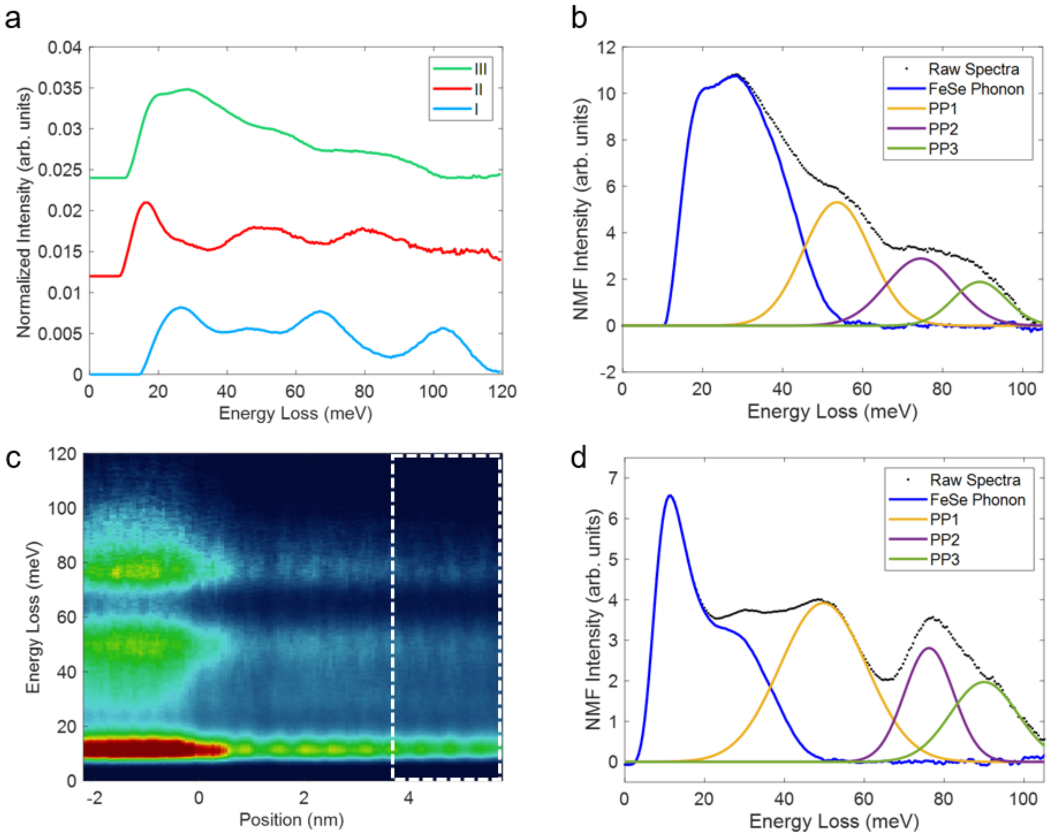

Fig. S4. The off-axis NMF spectra, on-axis EELS spectra and decomposition of phonon polariton. a. The NMF spectra for component I, II and III performed on the off-axis data. **b.** The gauss peak fitting for NMF component III spectrum. The spectral features above 45 meV are fitted by three gaussian peaks which are attributed to three branches of phonon polaritons (PPs). These signals were collected even in off-axis experimental geometry via multiple scattering, i.e., the high-energy electron undergoes inelastic scattering by PP and elastic scattering by Bragg scattering subsequently. The large overlap of diffraction disk in 35 mrad illumination enables this process easily. The peak energies are ~53 meV for PP1 (yellow), ~74 meV for PP2 (purple) and ~89 meV for PP3 (green). **c**. The line profile of atomically resolved on-axis EEL spectra across the interface. The spectral features above 45 meV extend to whole FeSe region without energy shift or significant intensity decay due to the delocalized nature of PPs. **d.** The gauss peak fitting for spectrum extracted from white dashed rectangle in **c**. Similar to **b**, three gaussian fitted PPs are located at energy ~50 meV (PP1), ~76 meV (PP2), and ~90 meV (PP3).

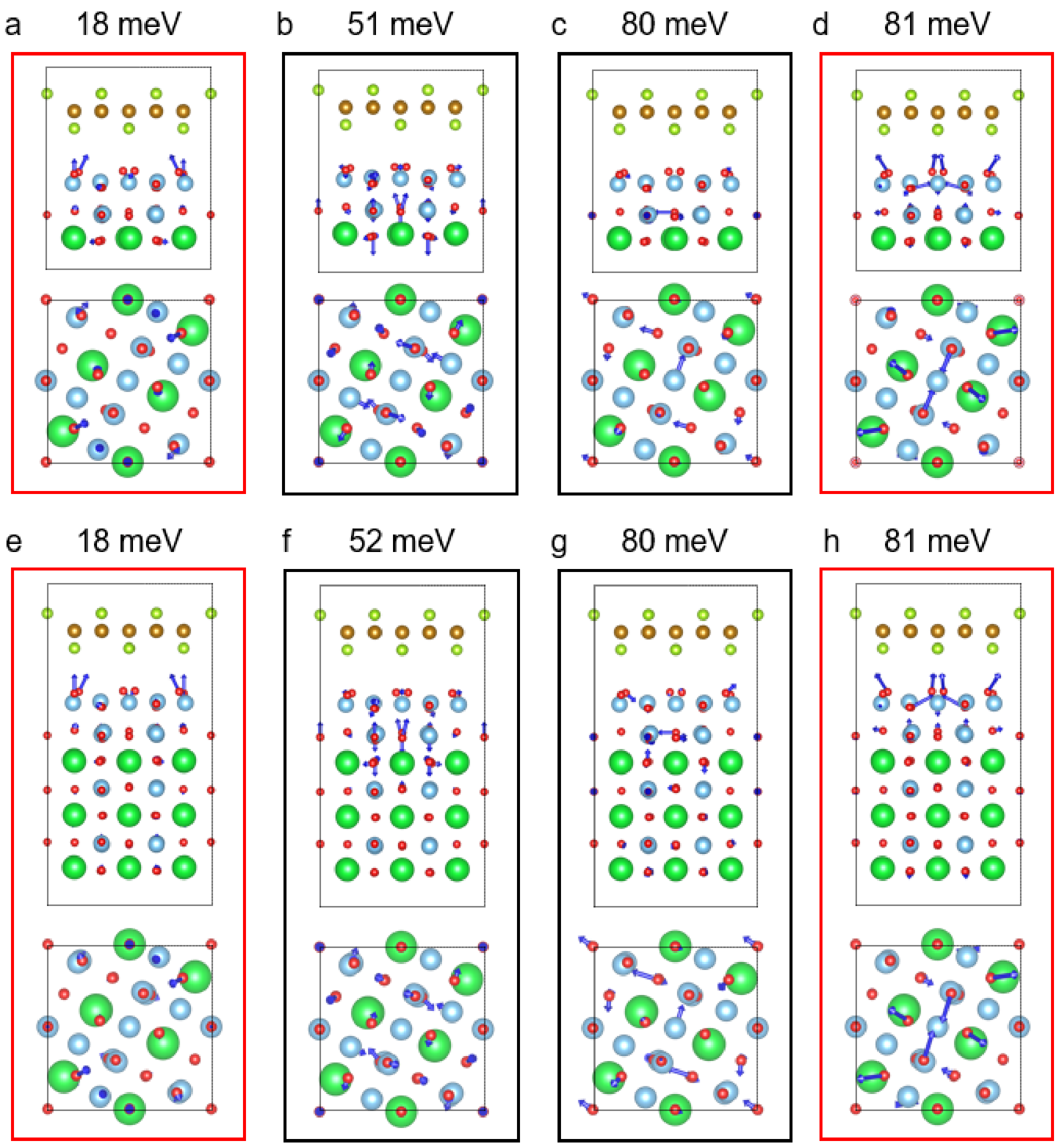

Fig. S5. The phonon eigenvectors of interfacial modes for FeSe/$SrTiO_3$ interface. a-d. The side view (upper panel) and top view (lower panel) of phonon eigenvectors in 1 UC interface structure with energy ~18 meV (**a**), ~51 meV (**b**), ~80 meV (**c**) and ~81 meV (**d**). **e-h.** The corresponding phonon eigenvectors in 3 UC interface structure with energy ~18 meV (**e**), ~52 meV (**f**), ~80 meV (**g**) and ~81 meV (**h**). The colors of the boxes correspond to the colors of the black and red arrows in Fig. 3a. These modes involve the vibrations much stronger at either Ti-O(B) or Ti-O(T) layer in both interface models, indicating their highly localized nature.

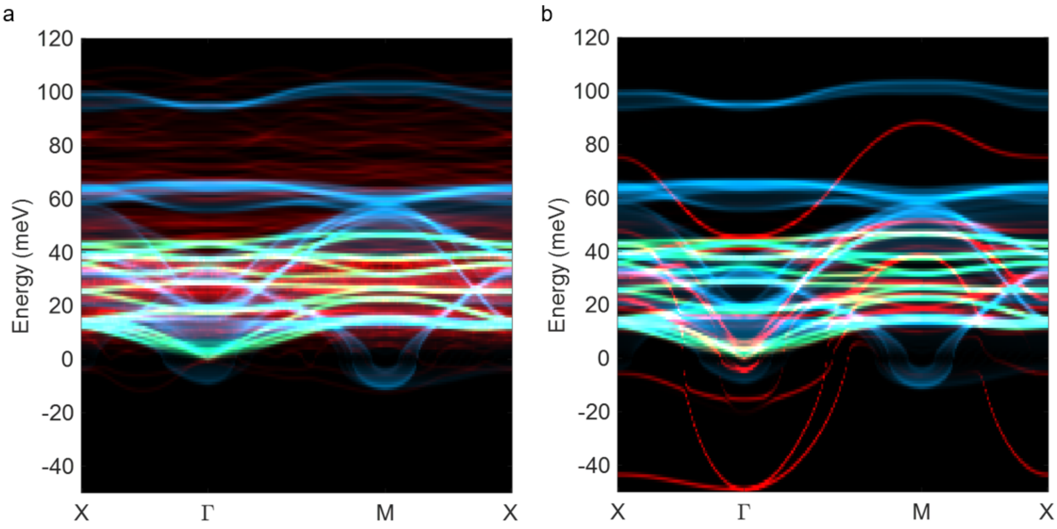

Fig. S6. The projected phonon dispersion of bulk $SrTiO_3$, bulk FeSe and FeSe/$SrTiO_3$ interface. The green and blue lines are phonon dispersion in bulk FeSe and bulk $SrTiO_3$ respectively, projected along [001] direction. The red lines are phonon dispersion projected onto interfacial Ti-O atoms in **a.** $\sqrt{5} \times \sqrt{5}\,R26.6°$ reconstructed 1 UC interface model and **b.** reconstruction-free 3 UC model. The red lines in **a** are flat, and lying outside the projected bands of bulk materials, further confirming the localized nature of interfacial phonons. Besides, no interfacial phonon with imaginary frequency is found at Γ point and only one interfacial phonon with no more imaginary frequency than that of bulk $SrTiO_3$ is found at other points in momentum space in **a**. On the contrary, several interfacial phonons with very large imaginary frequencies at Γ point are found in **b.** This indicates the interface with $\sqrt{5} \times \sqrt{5}\,R26.6°$ reconstruction is dynamically more stable than the reconstruction-free one.

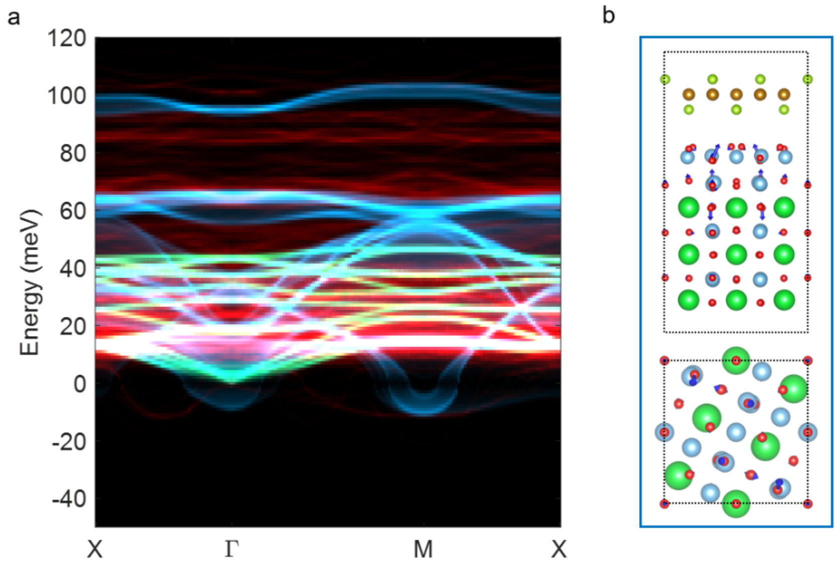

Fig. S7. The projected phonon dispersion and eigenvectors of interfacial modes in FeSe (1 UC)/$SrTiO_3$(3 UC) interface model. a. The green and blue lines are phonon dispersion in bulk FeSe and bulk $SrTiO_3$ respectively, projected along [001] direction. The red lines are phonon dispersion projected onto interfacial Ti-O atoms. **b.** The side view (upper panel) and top view (lower panel) of phonon eigenvectors corresponding to the mode in Fig. 3c. The dispersion in which red lines are lying outside the projected bands of bulk materials, and the phonon eigenvector only involving atoms of the top layer of $SrTiO_3$ help confirm the localized nature of interfacial phonons.

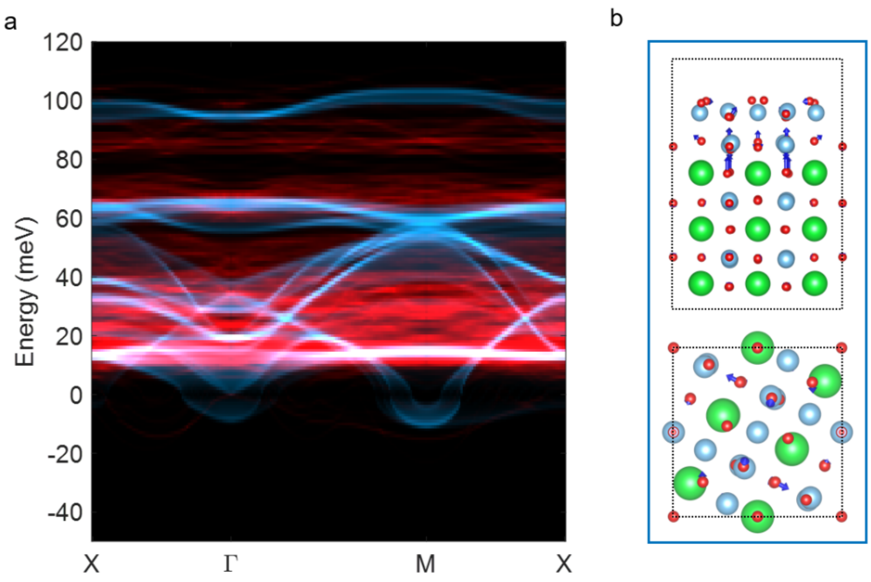

Fig. S8. The projected phonon dispersion and eigenvectors of interfacial modes in 3 UC $SrTiO_3$ surface model. a. The blue lines are phonon dispersion in bulk $SrTiO_3$ projected along [001] direction. The red lines are phonon dispersion projected onto surface Ti-O atoms. **b.** The side view (upper panel) and top view (lower panel) of phonon eigenvectors corresponding to the mode in Fig. 3c. This mode has an energy of ~93 meV, which is about 10 meV higher than the energy of the SCI mode. The pronounced softening in energy for the same vibrational mode can solely be attributed to the presence of FeSe.
