# Supplementary material for: Atomic-scale observation of localized phonons at FeSe/SrTiO3 interface
Source: Nat Commun. 2024 Apr 23;15:3418. doi: 10.1038/s41467-024-47688-5 (PMC11039677; doi:10.1038/s41467-024-47688-5)
Supplement: Supplementary file 2 — Peer Review File [file 41467_2024_47688_MOESM2_ESM.pdf]

nature portfolio

Peer Review File

Atomic-scale observation of localized phonons at FeSe/$SrTiO_3$ interface

Open Access This file is licensed under a Creative Commons Attribution 4.0 International License, which permits use, sharing, adaptation, distribution and reproduction in any medium or format, as long as you give appropriate credit to the original author(s) and the source, provide a link to the Creative Commons license, and indicate if changes were made. In the cases where the authors are anonymous, such as is the case for the reports of anonymous peer reviewers, author attribution should be to 'Anonymous Referee' followed by a clear attribution to the source work. The images or other third party material in this file are included in the article's Creative Commons license, unless indicated otherwise in a credit line to the material. If material is not included in the article's Creative Commons license and your intended use is not permitted by statutory regulation or exceeds the permitted use, you will need to obtain permission directly from the copyright holder. To view a copy of this license, visit http://creativecommons.org/licenses/by/4.0/.

Editorial Note: This manuscript has been previously reviewed at another journal that is not operating a transparent peer review scheme. This document only contains reviewer comments and rebuttal letters for versions considered at *Nature Communications*.

REVIEWER COMMENTS

Reviewer #1 (Remarks to the Author):
I have examined the revised manuscript and the author's response to the original referee reports. Overall, the authors have done an excellent job responding to many of my comments from the first round of review at Nature Materials. However, the connection between the observed interfacial mode and superconductivity in the FeSe monolayer is tenuous. In response to my comments, the authors provided additional DFT calculations for the interfacial mode and the resulting electron-phonon coupling. These calculations form are the sole evidence connecting the observed modes to the enhancement of superconductivity, so they must be rigorously assessed.

In that spirit, I have the following comments:
1) The authors argue that the calculated coupling \lambda = 0.1 is consistent with prior e-ph coupling strength estimates for this system. Rademaker et al. estimated lambda_m = 0.20 to get the right spectral weight of the replica band, and Faeth et al. confirmed this number in prior ARPES measurements. However, the lambda examined in those previous works differs from what the authors calculated. The interfacial e-ph coupling in FeSe/STO has to be strongly peaked in the forward scattering direction. (This peak is needed to make the interfacial mode attractive in the unconventional pairing channel expected in FeSe and produce the replica bands.) The lambda value computed by the authors is obtained by the integral of the Eliashberg function \alpha^2F, which is, in turn, calculated by a Fermi surface average of coupling constant |g(k,q)|^2. It thus represents an average coupling on the Fermi surface. The lambda value quoted by Faeth et al. and Rademaker et al. refers to the mass enhancement at the Fermi level $\lambda_m = d\Sigma/d\omega$. These two quantities are different when g(k,q) has a large forward scattering peak! To obtain $\lambda_m = 0.2$ in FeSe using a forward scattering phonon, one needs $\lambda ~ 0.5-1$ because the interaction is so sharply peaked in momentum space. To link these observations, one should look at the momentum dependence of the phonon linewidth, which indicates the q-dependence of g(k,q). For example, what is the width of the forward scattering peak inferred from the phonon linewidth?

2) The phonon linewidth plots shown in Fig. 3b indicate a forward scattering peak. However, similar peaks have been found in previous DFT calculations [see Wang et al., Rev. B 93, 134513], also done with quantum expresso. There, the authors found that this forward scattering peak's width depended on the q-grid size and vanished as the q-grid was made denser and denser. Have the authors checked if their calculations are robust against denser q-grids?

3) STO/FeSe slab calculations incorrectly predict a Ti band crossing the Fermi level. This band will contribute to the calculation of lambda. How much of the alpha^2F plot comes from scattering within the Ti bands rather than the FeSe bands? Have the authors tried computing alpha^2F for their STO slab with the TiO2 double layer and no FeSe? Is it comparable to the results shown?

Again, given the importance of the DFT calculations to the primary conclusion, these points need to be addressed before I can provide a recommendation.

Reviewer #2 (Remarks to the Author):

The authors have made commendable efforts in integrating the suggestions and comments of the reviewers into the revised manuscript, resulting in a significant enhancement of the manuscript's overall quality. The incorporation of new experimental and theoretical data, along with the further analysis conducted, has made the manuscript more convincing. It is evident that the concerns raised by the reviewers have been aptly addressed, leading to a more robust and insightful study.
The key findings regarding the highly localized interface phonons and their significant role in providing electron-phonon coupling strength are very interesting. The additional phonon calculations for the SrTiO3 surface structure without FeSe enhance the manuscript's comprehensiveness, underscoring the authors'commitment to delivering a thorough and well-supported piece of research.
I recommend its publication.

Response to reviewers' comments

We would like to thank the two reviewers for their thorough and highly constructive comments concerning our manuscript. The reviewer#1 raised comments on the DFT calculation details, while reviewer#2 recommended publication. In response to the reviewer#1's comments and suggestions, we have provided new theoretical data, conducted further analysis, and thus enhanced our understanding and improved the manuscript. We believe that the concerns are addressed appropriately in the revised manuscript.

The revised manuscript highlights the presence of highly localized interfacial phonons at the FeSe/$SrTiO_3$ interface and the possible role in the interface superconducting for the first time. This coupling mechanism with short-range interaction is independent and complementary to the widely accepted long-range F-K phonons or optical mode of $SrTiO_3$ substrate, likely enhancing interfacial electron-phonon coupling collaboratively. These findings not only provide new insights into understanding the underlying mechanisms of enhanced superconductivity at interfaces, but also stimulates further theoretical experimental studies. We believe that our manuscript now holds broad appeal in areas of superconductivity, interface science and other significant areas in material science.

Major changes in the revision:

According to the reviewer#1's suggestions and comments, we have revised manuscript properly to improve the manuscript. Changes are highlighted with blue font color in the revised manuscript. Below is a list for major ones.

1. We have revised the description of the electron-phonon coupling constant for the strongly interacting interfacial mode. The manuscript now emphasizes its high coupling constant, indicating its significant contribution in the interface enhancement of electron-phonon coupling.

2. We have added discussion on the limitation of our calculation and experiments, which will likely stimulate further studies in future.

3. We have made better clarification in the discussion by emphasizing the new coupling mechanism provided by short-range interfacial mode complement rather than contradict commonly accepted long-range F-K phonons. This result provides new insights into understanding the underlying mechanisms of enhanced superconductivity at interfaces.

4. Other changes following reviewer’s suggestions. Please see our response below for detail.

Point-by-point response:

Response to reviewer 1

REVIEWER COMMENTS

Reviewer #1 (Remarks to the Author):

I have examined the revised manuscript and the author's response to the original referee reports. Overall, the authors have done an excellent job responding to many of my comments from the first round of review at Nature Materials. However, the connection between the observed interfacial mode and superconductivity in the FeSe monolayer is tenuous. In response to my comments, the authors provided additional DFT calculations for the interfacial mode and the resulting electron-phonon coupling. These calculations form are the sole evidence connecting the observed modes to the enhancement of superconductivity, so they must be rigorously assessed.

Response: We thank the reviewer for the thorough examination of our manuscript. We understand the reviewer's concern on the "tenuous connection" between the observed interfacial mode and superconductivity in our work mainly because direct experimental evidence such as in situ transport measurement at low temperature is lacking, for which it's unfortunately impossible to be done with the current instruments. On the other hand, the theoretical calculations generally involving many simplifications, may not precisely reflect the practical situation, making the connection "tenuous". To mitigate the reviewer's concern, in this revised manuscript, we provided new theoretical data, conducted further analysis, and thus enhanced our understanding and improved the manuscript. The details are included below.

On the other hand, with this work we would like to emphasize that the electron-phonon coupling caused by the short-range interfacial phonon at FeSe/$SrTiO_3$ interface may also play a considerable role in the interfacial superconducting (as the density functional theory illustrates a strong coupling strength of interfacial mode), providing a new mechanism that is independent from the previously accepted optical modes of $SrTiO_3$ or F-K phonons with long-range interactions. Note that we do not intend to solely attribute all the superconductivity enhancement to the interfacial phonon. Instead, the short-range interfacial modes complement rather than contradict the previously identified long-range F-K phonons, thereby enhancing the electron-phonon coupling at the interface collaboratively.

Therefore, we believe this work not only provides new insights into understanding the underlying mechanisms of enhanced superconductivity at interfaces, but also stimulates further theoretical and experimental studies in future.

In that spirit, I have the following comments:

1) The authors argue that the calculated coupling \lambda = 0.1 is consistent with prior e-ph coupling strength estimates for this system. Rademaker et al. estimated lambda_m = 0.20 to get the right spectral weight of the replica band, and Faeth et al. confirmed this number in prior ARPES measurements. However, the lambda examined in those previous works differs from what the authors calculated. The interfacial e-ph coupling in FeSe/STO has to be strongly peaked in the forward scattering direction. (This peak is needed to make the interfacial mode attractive in the unconventional pairing channel expected in FeSe and produce the replica bands.) The lambda value computed by the authors is obtained by the integral of the Eliashberg function \alpha^2F, which is, in turn, calculated by a Fermi surface average of coupling constant |g(k,q)|^2. It thus represents an average coupling on the Fermi surface. The lambda value quoted by Faeth et al. and Rademaker et al. refers to the mass enhancement at the Fermi level $\lambda_m = d\Sigma/d\omega$. These two quantities are different when g(k,q) has a large forward scattering peak! To obtain $\lambda_m = 0.2$ in FeSe using a forward scattering phonon, one needs $\lambda ~ 0.5-1$ because the interaction is so sharply peaked in momentum space. To link these observations, one should look at the momentum dependence of the phonon linewidth, which indicates the q-dependence of g(k,q). For example, what is the width of the forward scattering peak inferred from the phonon linewidth?

Response: We really appreciate the reviewer for pointing out the difference between conventional electron-phonon coupling constant λ and mass enhancement λ_m in the previous work. In the revised manuscript, we have added clarification on such difference and modified the statement.

According to the reviewer's suggestion, we have plotted the momentum dependence of phonon linewidth of strongly coupling interfacial (SCI) mode, as shown in Fig. R1. The phonon linewidth decays fast as momentum increase, indicating its forward scattering behavior. The decay length $q_0 \approx 0.5/a$, results in a mass enhancement $\lambda_m \approx 0.04$ due to SCI mode alone[1]. Although this value is relatively small, it contributes a high coupling strength compared with other modes including previously considered optical

modes of $SrTiO_3$[2] (also shown in Fig. 3b). Furthermore, we would to point out that the SCI mode complements rather than contradicts to the previously identified optical phonons of $SrTiO_3$ and F-K phonons, thereby enhancing the electron-phonon coupling at the interface collaboratively. Some other effect including charge transfer[3], magnetic effect[4] or correlation[5] may further enhance the coupling strength, which requires further investigations but is beyond the scope of this study.

Accordingly, we have revised our description of in the manuscript page 8 line 214-218 to be:

"*From the Eliashberg spectral function curves, we obtain the electron-phonon coupling constant of the SCI phonon mode as $\lambda \approx 0.10$. This suggests that interfacial modes may play a significant role in the interfacial electron-phonon coupling, in complementary to the previously considered optical modes of $SrTiO_3$ or F-K phonons.*"

We have also made further clarifications in the discussion page 9 line 250-252:

"*Furthermore, some other effects such as magnetic effect, charge transfer or correlation that may further enhance the coupling strength, are also needed to be considered in the calculations.*"

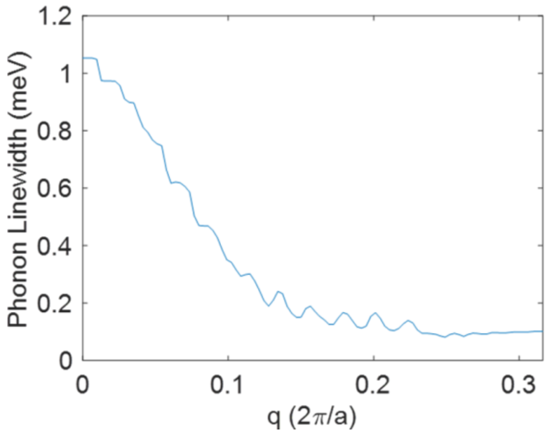

Figure R1 | The momentum dependence of phonon linewidth of the strongly coupling interfacial mode | The phonon linewidth decays fast as momentum increases, indicating its forward scattering behavior. The decay length $q_0 \approx 0.5/a$.

2) The phonon linewidth plots shown in Fig. 3b indicate a forward scattering peak. However, similar peaks have been found in previous DFT calculations [see Wang et al., Rev. B 93, 134513], also done with quantum expresso. There, the authors found that this forward scattering peak's width depended on the q-grid size and vanished as the q-

grid was made denser and denser. Have the authors checked if their calculations are robust against denser q-grids?

Response: We thank the reviewer for pointing this out. We note that our structure is $\sqrt{5}\times\sqrt{5}$ R26.6° reconstructed, which means that the q-grid is already $\sqrt{5}\times\sqrt{5}$ meshed. We also agree that a denser q-grid is needed to accurately calculate the coupling strength especially in the case of forward scattering. However, our structure contains 60 atoms, and the calculation with a denser q-grid would require more than 2 months of computation time and 2 terabytes of storage space, which is beyond the capability of the best computational resources we can access (High-performance Computing Platform of Peking University).

In this sense, we agree with the reviewer that the calculated electron-phonon coupling strength of the SCI mode may be overestimated. To clarify this, we have cited the relevant research and added more discussion in the revised manuscript page 9 line 244-249

"*It should be noted, however, that there are some limitations to our present study and further investigations are needed in future to more precisely correlate the localized interfacial phonons with the superconductivity property. For the calculation, although our model is already* $\sqrt{5}\times\sqrt{5}$ R26.6° *reconstructed, the calculated electron-phonon coupling strength of the SCI mode is likely overestimated. To more accurately estimate the electron-phonon coupling strength, an even denser q-grid is probably required.*"

3) STO/FeSe slab calculations incorrectly predict a Ti band crossing the Fermi level. This band will contribute to the calculation of lambda. How much of the alpha^2F plot comes from scattering within the Ti bands rather than the FeSe bands? Have the authors tried computing alpha^2F for their STO slab with the TiO2 double layer and no FeSe? Is it comparable to the results shown?

Response： We greatly appreciate the reviewer's valuable suggestion. We have calculated the projected electron density of states (EDOS) of our model, shown in Fig. R2. There indeed is contribution from Ti bands at Fermi level, however, the EDOS of Ti bands is less than 1/10 of Fe bands. Moreover, we have made further calculations of the Eliashberg spectral function and electron-phonon coupling constant in the $\sqrt{5}\times\sqrt{5}$ R26.6° reconstructed $SrTiO_3$ slab with Ti-O double layer (same structure in the manuscript but without FeSe layer) according to the reviewer's suggestion. It turns

out that the Eliashberg spectral function $\alpha^2 F$ is all zero. Thus, we conclude that the electron-phonon coupling in our system is mainly contributed by the coupling from FeSe electrons.

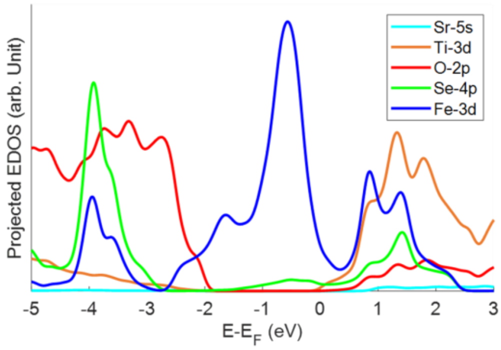

Figure R2 | The Project Electron Density of States at FeSe/SrTiO$_3$ Interface | The Electron density of states projected to Sr-5d (cyan), Ti-3d (orange), O-2p (red), Se-4p(green) and Fe-3d(blue) orbit. The projected EDOS of Ti band is less than 1/10 of Fe band at the Fermi level.

Again, given the importance of the DFT calculations to the primary conclusion, these points need to be addressed before I can provide a recommendation.

Response: We thank the reviewer again for the rigorous examination of our manuscript. With the above response, we believe that DFT calculation is strengthened and the reviewer’s comments are appropriately addressed.

Reviewer #2 (Remarks to the Author):

The authors have made commendable efforts in integrating the suggestions and comments of the reviewers into the revised manuscript, resulting in a significant enhancement of the manuscript's overall quality. The incorporation of new experimental and theoretical data, along with the further analysis conducted, has made the manuscript more convincing. It is evident that the concerns raised by the reviewers have been aptly addressed, leading to a more robust and insightful study.
The key findings regarding the highly localized interface phonons and their significant role in providing electron-phonon coupling strength are very interesting. The additional

phonon calculations for the SrTiO3 surface structure without FeSe enhance the manuscript's comprehensiveness, underscoring the authors' commitment to delivering a thorough and well-supported piece of research.
I recommend its publication.

Response: We would like to thank the reviewer for the recommendation for the publication and positive comments on the importance of our work.

Reference

1. Wang, Y., Nakatsukasa, K., Rademaker, L., Berlijn, T. & Johnston, S. Aspects of electron–phonon interactions with strong forward scattering in FeSe Thin Films on $SrTiO_3$ substrates. *Supercond. Sci. Technol.* **29**, 054009 (2016).
2. Wang, Y., Linscheid, A., Berlijn, T. & Johnston, S. *Ab initio* study of cross-interface electron-phonon couplings in FeSe thin films on $SrTiO_3$ and $BaTiO_3$. *Phys. Rev. B* **93**, 134513 (2016).
3. Zhou, Y. & Millis, A. J. Charge transfer and electron-phonon coupling in monolayer FeSe on Nb-doped $SrTiO_3$. *Phys. Rev. B* **93**, 224506 (2016).
4. Li, B., Xing, Z. W., Huang, G. Q. & Xing, D. Y. Electron-phonon coupling enhanced by the FeSe/$SrTiO_3$ interface. *Journal of Applied Physics* **115**, 193907 (2014).
5. Kulić, M. L. & Zeyher, R. Influence of strong electron correlations on the electron-phonon coupling in high- T c oxides. *Phys. Rev. B* **49**, 4395–4398 (1994).

REVIEWERS' COMMENTS

Reviewer #1 (Remarks to the Author):

I want to thank the authors for their additional calculations and willingness to consider my comments. The current version of the manuscript has addressed most of my comments to the best of what can reasonably be expected. I would still like to see some analysis of what is going on with the forward scattering peak as a function of q, but that is probably beyond the scope of this work.

I recommend publication at this time.

Response to reviewers' comments

We would like to thank two reviewers for taking the time and effort necessary to review our manuscript, and we appreciate their positive feedback.

Point-by-point response:

Response to reviewer 1

Reviewer #1 (Remarks to the Author):

I want to thank the authors for their additional calculations and willingness to consider my comments. The current version of the manuscript has addressed most of my comments to the best of what can reasonably be expected. I would still like to see some analysis of what is going on with the forward scattering peak as a function of q, but that is probably beyond the scope of this work.

I recommend publication at this time.

Response: We would like to thank the reviewer for the recommendation for the publication and positive comments on our work. Indeed, the analysis of forward scattering peak as a function of q is of great importance in FeSe/$SrTiO_3$ interface. However, this analysis is beyond the scope of this work.
